# Surveying Mercury Levels in Hair, Blood and Urine of under 7-Year Old Children from a Coastal City in China

**DOI:** 10.3390/ijerph111112029

**Published:** 2014-11-20

**Authors:** Guixia Chen, Xiaoxin Chen, Chonghuai Yan, Xingdong Wu, Guozhang Zeng

**Affiliations:** 1 Xiamen Maternity and Child Health Care Hospital, Xiamen 361000, China; 2 School of Life Sciences, Xiamen University, Xiamen 361005, China; 3 Shanghai Key Laboratory of Children’s Environmental Health, School of Medicine, Shanghai Jiaotong University Shanghai 200092, China

**Keywords:** mercury, children, blood, urine, hair

## Abstract

*Aim*: The average mercury load in children under 7-years old was determined in a populated but not overly industrial coastal area in China. *Methods*: 395 blood samples, 1072 urine samples, and 581 hair samples were collected from 1076 children, aged 0 to 6 years, from eight representative communities of Xiamen, China. Mercury levels in the samples were surveyed. *Results*: The 95% upper limits of mercury in blood, urine, and hair for the children were 2.30, 1.50 and 2100.00 μg/kg, respectively. Levels tended to increase with age. Correlation analyses showed that mercury levels in blood and urine correlated with those in hair (*n* = 132), *r* = 0.49, *p* < 0.0001 and *r* = 0.20, *p* = 0.0008; however, blood mercury levels did not correlate with urine levels (*n* = 284), *r* = 0.07, *p* = 0.35. *Conclusions*: Surveying the average mercury load in children 0 to 6 years, and the 95% upper limit value of mercury in their blood, urine, and hair should help guide risk assessment and health management for children.

## 1. Introduction

Mercury (Hg) is ubiquitous in the global environment and derives from both natural sources and human enterprise [1]. Environmental exposures to Hg contribute to a wide range of problems, e.g., inattention, memory disturbance, learning problems, impairment of social behavior, and low IQ [2]. These toxic effects are most serious in the developing central nervous system of children, so the greatest concern surrounds prenatal and early childhood Hg exposure [3].

In environmental medicine, human bio-monitoring is a key method for assessing and evaluating the level of internal exposure to environmental pollutants experienced by both populations and individuals [4]. For a specified population in a defined state of health a clinical laboratory empirically establishes reference values (RVs) to identify subjects with elevated and potentially dangerous levels of exposure [5]. The 95th percentile determines the RV, indicating the upper margin of background exposure to a given environmental toxin at a given time. RVs should be revised periodically, to reflect changes in environmental pollutants [6], particularly persistent organic pollutants, trace elements, and insecticides [7,8].

Past surveys of methyl Hg exposure in humans have measured organic Hg content in blood and hair samples, while urinalysis was used to measure excreted, inorganic Hg [9,10,11,12,13,14]. Recently, new methods have been devised for accurately measuring Hg levels in hair samples [14]. Hair sampling might offer an alternative to urine and blood sampling, since hair sampling can reflect long-term Hg exposure and has the merits of high stability and low invasiveness [13]. Despite these advantages, hair analysis is suspected of having unique limitations, such as inter-individual variability and surface contamination [15].

To establish RV values and also compare hair sampling for determining Hg exposure, we collected and compared samples of blood, urine, and hair from a population of children and compared the relative levels with each type of sample. The population we analyzed included children, aged 0 to 6 years in Xiamen, China. We chose this location because it is relatively unremarkable in environmental contamination, being neither highly industrialized nor rural, and might be representative of many other areas where children live. For our sample, correlation analyses among Hg levels in blood, urine, and hair were also investigated.

## 2. Materials and Methods

### 2.1. Subject Selection

Subjects were children, selected through a stratified cluster design from eight representative communities in Xiamen, a coastal city located in southern China. Xiamen is situated at latitude 24°26'46"N and longitude 118°04'04"E, which contains Xiamen Island, Gulangyu Island, and part of the rugged mainland coastal region from the left bank of the Jiulong River in the west, to the islands of Xiang’an in the northeast; the area covers 1699.39 square kilometers that holds a population of 3.67 million. Samples were collected from 1076 healthy children, with more than 100 children chosen from each Xiamen community.

Informed consent was obtained from each survey participant, as well as from the parents and schoolteachers. Before the samples were collected, participants filled out a questionnaire about individual and socioeconomic characteristics and medical history. The study population we selected was a typical group of children living in Xiamen, who were neither believed to have been exposed to toxic substances nor to be living in unusual conditions. None of the children included in the study reported having any diseases.

### 2.2. Sample Collection

We obtained 395 blood samples by vein puncture and collected blood in 10-mL metal-free, evacuated, blood-collection tubes, which contained 0.05 mL of 15% EDTA K3 solution (7.5 mg). The tubes had a silicon-lubricated stopper but no interior coating. One blank container was included each day for contamination checks. Samples were refrigerated at approximately −20 °C until thawed for processing.

We collected 1072 hair samples from the nape of the neck, as near to the scalp as possible, using stainless steel scissors. Samples were sealed separately in labeled polyethylene zip-lock bags and not opened until cleaned and processed in the laboratory. In total, 581 hair sample specimens were collected from eight communities. Hair samples were washed with water and acetone according to the method described by Carneiro *et al.* [16]. After washing, samples were dried in a class-100 laminar-flow hood, and then cut into less than 0.5 cm lengths before analysis.

The 1072, morning, spot-urine samples were collected in polypropylene sampling vessels and stored at −20 °C prior to analysis. To collect samples from small infants, we attached disposable plastic urine bags, which stick to the outside of the urethral orifice and pack inside their diapers.

### 2.3. Mercury Analysis

Total Hg levels in blood, hair, and urine were determined with a Direct Hg Analyzer (DMA-80, Milestone srl, Rome, Italy) [17], which does not require sample preparation or other wet chemistry prior to the analysis. The Analyzer is matrix-independent and can analyze solid, aqueous, and gas samples with equal efficiency. Samples are carried to a catalyst by oxygen flow, oxidized, and then halogens and nitrogen/sulphur oxides are trapped. The final decomposition products are then passed through an Hg amalgamator, which collects Hg. The Hg amalgamator is heated to 700 °C and Hg released and quantified. The DMA-80 achieves a detection limit as low as 0.001 nanograms of Hg and is capable of measuring up to 30,000 nanograms of Hg, equivalent to a concentration of 300 mg/kg (300 ppm) in a 100 mg sample. All samples were prepared in triplicate. To ensure the accuracy of the analytical methods and results, quality control (QC) samples (International Stardard, GBW 080124) were analyzed in parallel. Total Hg levels were calculated as micrograms per litre in blood and urine, and as microgram per kilogram in hair. For urinalysis, we collected spot urine in the morning. We did not normalize to urinary creatinine. The sample testing was finished in five years.

### 2.4. Statistical Analysis

The descriptive statistical parameters (arithmetic mean, standard deviation, minimum, maximum, median, percentiles, geometric mean [GM]) and 95% confidence intervals were calculated for the Hg levels of blood, urine, and hair. The Mann-Whitney U test was used to evaluate statistical differences in Hg levels between genders. The associations among Hg levels in blood, hair, and urine samples were tested using Spearman correlation analyses. The MelCalc Software, version 12.7.0.0, was used for calculations (MedCalc Software bvba, Mariakerke, Belgium). Statistical significance was set at *p* < 0.05. The tests revealed that the samples from one child contained Hg at levels greater than five standard deviations above the mean. This child was excluded from the statistical analysis.

## 3. Results

### 3.1. Distribution of Mercury Levels in Blood, Urine, and Hair 

**Figure 1:**
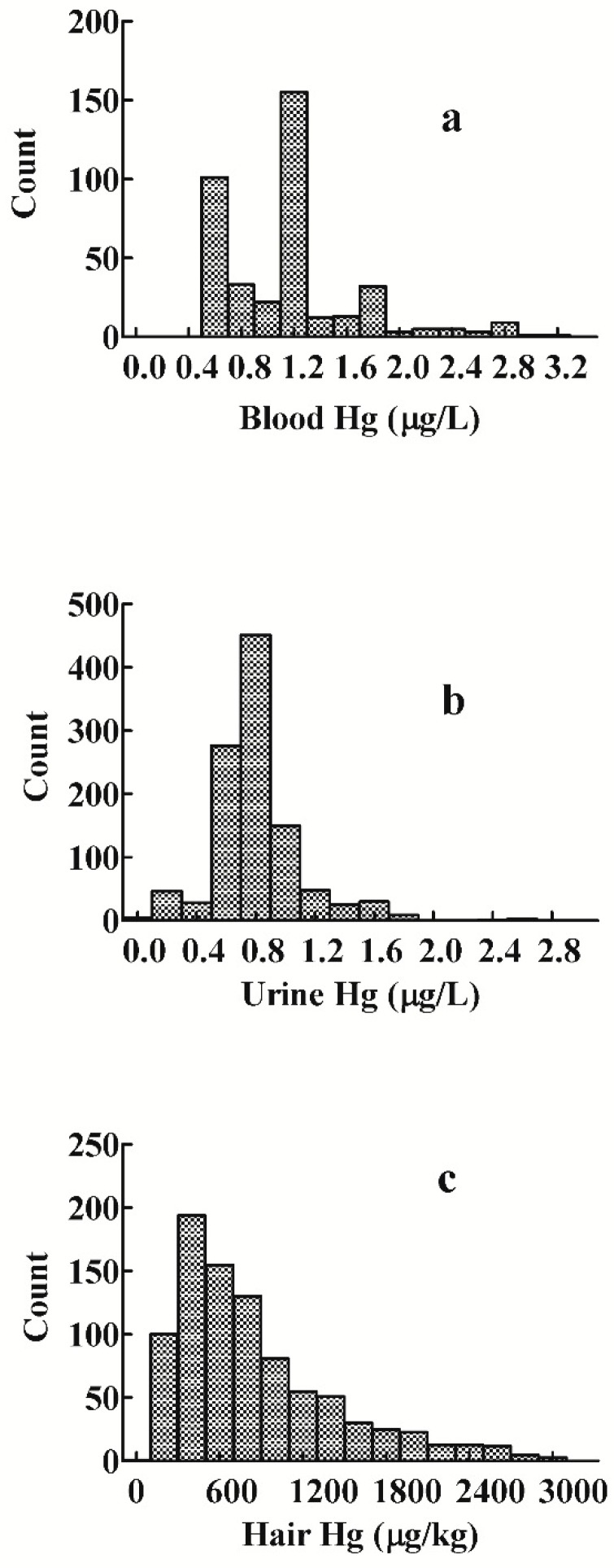
Distribution of mercury levels for children in Xiamen, China: (**a**) blood analysis: *n* = 395; (**b**) urine analysis: *n* = 1072; (**c**) hair analysis: *n* = 581.

The distribution of Hg levels in the 395 blood, 1072 urine, and 581 hair samples revealed a right-skewed pattern (Figure 1a–c). The frequency distribution pattern of Hg level in urine was close to log-normal (Figure 1b).

### 3.2. Blood Mercury Levels

As shown in Table 1, Hg levels in the children’s blood ranged from 0.56 to 3.15 μg/L (GM 1.05 μg/L, median 1.13 μg/L), with the 95% percentile at 2.28 μg/L and the 95th percentile confidence interval at 1.84 to 2.64 μg/L. Thus, in Xiamen, China, the RV of blood Hg was 2.30 μg/L for children aged 0 to 6 years. In boys, the blood Hg level showed no statistically significant difference from that in girls (GM 1.05 μg/L, GM-CI, 0.99 to 1.12 μg/L in boys *vs.* GM 1.04 μg/L, GM-CI, 0.98 to 1.10 μg/L in girls. Blood Hg level did increase with advancing age, since children aged 0 to 1 years, 2 to 3 years, and 4 to 6 years, respectively, showed GMs of 0.97, 1.00, and 1.06 μg/L (Table 2).

### 3.3. Urine Mercury Levels

**Figure 2:**
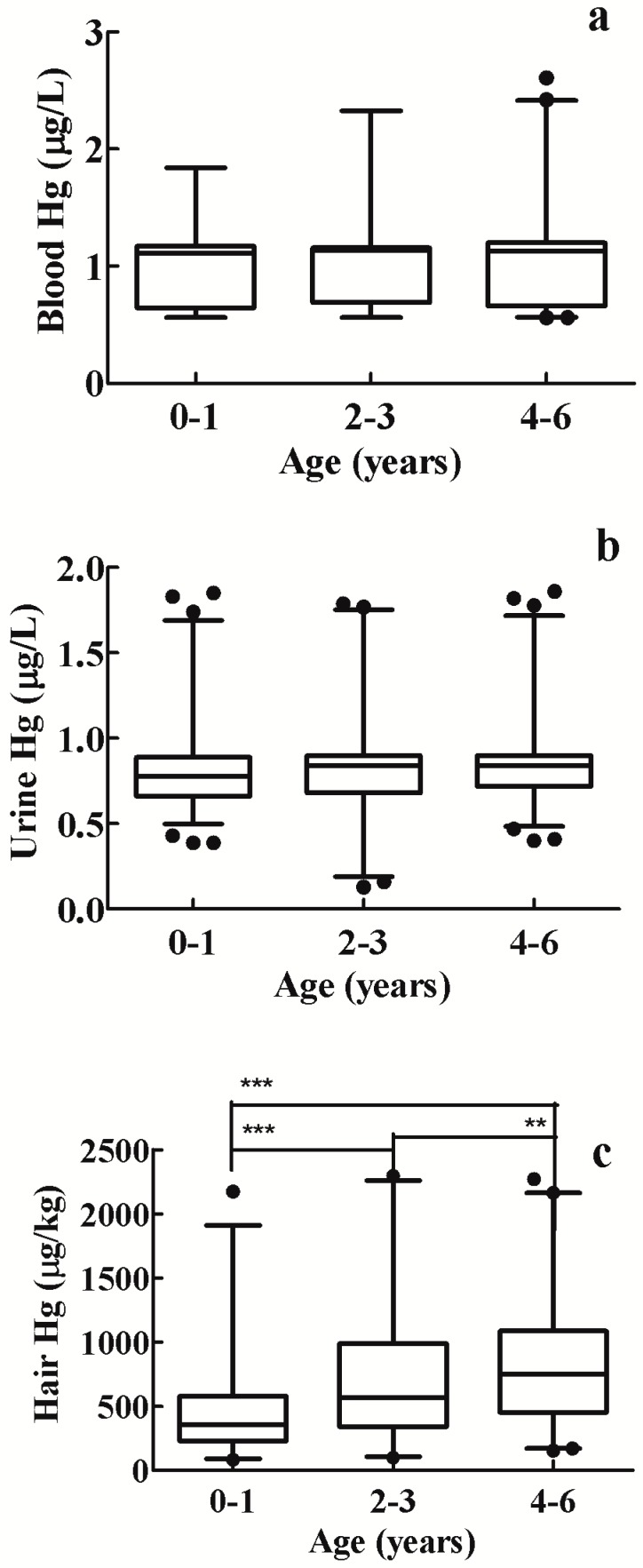
Box and whisker plots display the distributions of mercury levels in children in Xiamen, China (●outlier); (**a**), (**b**), and (**c**) show blood, urine and hair mercury levels of different age groups in 2006.

Notes: ** denotes a significant difference at *p* < 0.001 (*t*-test); *** denotes a significant difference at *p* < 0.0001 (*t*-test).

The Hg content in urine ranged from 0.03 to 2.63 μg/L (GM 0.75 μg/L, median 0.83 μg /L). The 95% percentile was 1.42 μg/L, the 95th percentile confidence interval from 1.31 to 1.51 μg/L (Table 1). Thus, the RV of urine Hg in this sample of children was 1.50 μg /L. Like with the blood Hg levels, urine Hg in boys (GM 0.77 μg/L, GM-CI 0.75–0.80 μg/L) showed no statistically significant difference from that in girls (GM, 0.73 μg/L, GM-CI 0.70–0.76 μg/L; Table 3). As with the blood analysis, Hg levels in urine increased with children’s age (Table 2), since levels in the 4 to 6-year age group averaged significantly higher than that in the 0 to 1-year age group (*p* = 0.0096, Mann-Whitney U test; Figure 2b).

**Table 1:** Mercury levels in children’s blood, urine, and hair.

Sample	n	Range	x ± SD	Median	GM ^a^	CI-CM ^b^	P95 ^c^	CI P 95 ^d^	RVs
Blood (μg/L)	395	0.56–3.15	1.14 ± 0.51	1.13	1.05	1.00−1.09	2.28	1.84−2.64	2.30
Urine (μg/L)	1072	0.03–2.63	0.82 ± 0.30	0.83	0.75	0.73−0.77	1.42	1.31−1.51	1.50
Hair (μg/kg)	581	99.0–3280.0	748.2 ± 598.4	550.0	569.8	536.7−604.9	2060.0	1900.4−2310.2	2100.0

**^a^** Geometric mean; **^b^** 95% confidence interval for GM; **^c^** 95th percentile; **^d^** CI P 95 confidence interval of P 95.

**Table 2:** Comparison of mercury concentrations in blood, urine, and hair of children aged 0 to 6 years.

Sample	n	Range	x ± SD	Median	GM ^a^	CI-CM ^b^	P95 ^c^	CI P 95 ^d^	RVs
Blood									
0–1 years (μg/L)	62	0.56–2.61	1.05 ± 0.42	1.11	0.97	0.88–1.08	1.77	—	1.80
2–3 years (μg/L)	82	0.56–2.83	1.07 ± 0.39	1.14	1.00	0.93–1.08	1.75	1.39–2.70	1.80
4–6 years (μg/L)	251	0.56–3.15	1.16 ± 0.53	1.14	1.06	1.00–1.12	2.30	2.00–2.75	2.30
Urine									
0–1 years (μg/L)	425	0.03–2.63	0.79 ± 0.29	0.75	0.73	0.70–0.76	1.35	1.25–1.48	1.40
2–3 years (μg/L)	252	0.10–1.79	0.82 ± 0.31	0.83	0.75	0.71–0.79	1.51	1.30–1.67	1.50
4–6 years (μg/L)	395	0.13–2.55	0.84 ± 0.30	0.83	0.78	0.75–0.81	1.46	1.23–1.65	1.50
Hair									
0–1 years (μg/kg)	195	116.0–3259.0	483.6 ± 390.2	363.0	388.0	354.9–424.2	1173.0	995.2–1523.7	1200.0
2–3 years (μg/kg)	172	99.0–2932.0	809.2 ± 625.3	602.5	619.0	554.4–691.2	2195.0	1949.7–2643.3	2200.0
4–6 years (μg/kg)	214	153.0–3280.0	939.4 ± 647.8	768.0	752.9	687.4–824.5	2466.0	2022.5–2725.7	2500.0

**^a^** Geometric mean; **^b^** 95% confidence interval for GM; **^c^** 95th percentile; **^d^** CI P 95 confidence interval of P 95.

**Table 3:** Mercury levels in blood, urine, and hair of boys and girls of children aged 0 to 6 years.

Sample	n	Range	x ± SD	Median	GM ^a^	CI-CM ^b^	P95 ^c^	CI P 95 ^d^	RVs
Blood									
Boys (μg/L)	214	0.56−3.15	1.16 ± 0.56	1.13	1.05	0.99−1.12	2.68	2.17−2.80	2.70
Girls (μg/L)	181	0.56−2.90	1.11 ± 0.44	1.14	1.04	0.98−1.10	1.84	1.74−2.27	1.90
Urine									
Boys (μg/L)	606	0.04−2.63	0.83 ± 0.31	0.83	0.77	0.74−0.80	1.50	1.39−1.63	1.50
Girls (μg/L)	466	0.03−2.55	0.79 ± 0.28	0.82	0.73	0.70−0.76	1.30	1.19−1.47	1.30
Hair									
Boys (μg/kg)	308	116.0−3272.0	746.6 ± 596.9	570.5	572.5	528.0−620.7	2165.0	1755.6−2573.3	2200.0
Girls (μg/kg)	273	99.0−3280.0	750.1 ± 601.2	531.0	566.8	518.5−619.5	2051.0	1762.7−2473.2	2100.0

**^a^** Geometric mean; **^b^** 95% confidence interval for GM; **^c^** 95th percentile; **^d^** CI P 95 confidence interval of P 95.

### 3.4. Hair Mercury Levels

The RV level for the group’s hair was 2100.0 μg/kg (Figure 1). As with the measurements in blood and urine, the hair of boys (Table 2) showed no statistically significant difference from that of girls. Children aged 4 to 6 years had significantly higher RVs compared to children aged 0 to 1 years (*p* < 0.0001, Mann-Whitney U test), and 2 to 3 years (*p* = 0.0069, Mann-Whitney U test; Figure 2c).

### 3.5. Correlation between Blood, Urine, and Hair Mercury Levels in Children 

**Figure 3:**
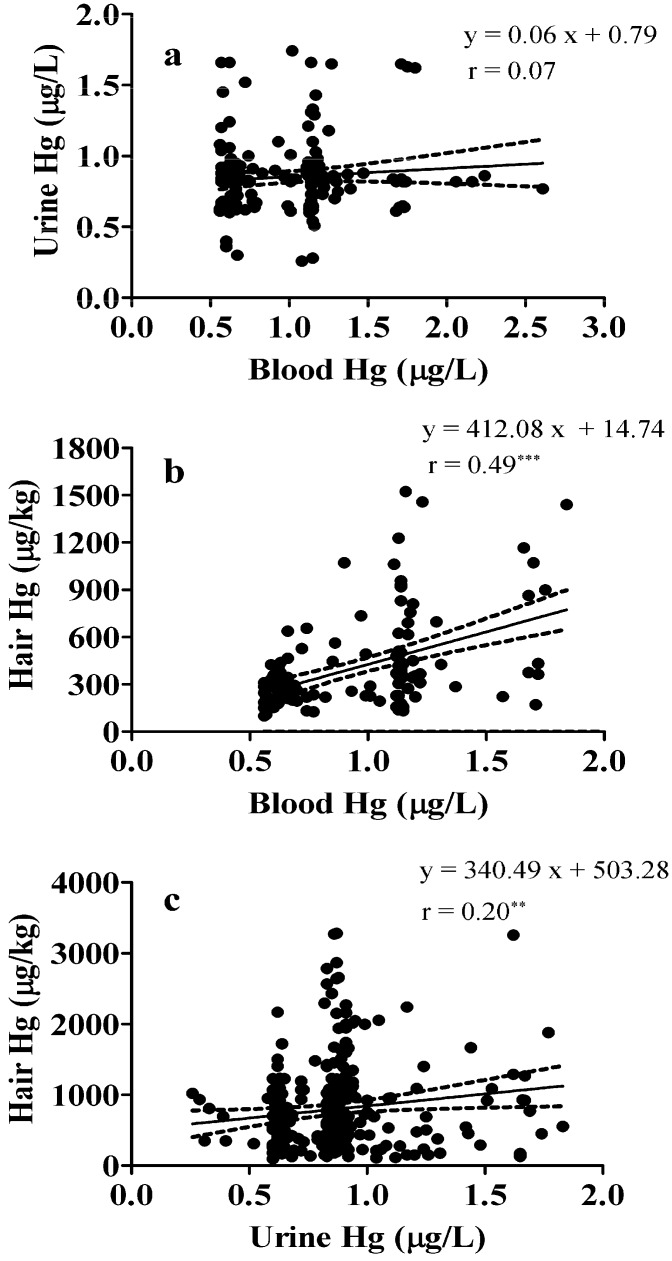
Correlations between (**a**) blood and urine mercury levels, (**b**) blood and hair mercury levels and (**c**) urine and hair mercury levels of children aged 0 to 6 years in Xiamen, China.

Notes: *n* = 171 for blood and urine, *n* = 132 for blood and hair, and *n* = 284 for urine and hair; ** *p* < 0.001, *** *p* < 0.0001; dotted lines denote 95% confidence intervals for the regression line.

The correlations between blood, urine, and hair Hg contents are given in Figure 3, showing that among the three groups, blood and hair measurements correlated best (*n* = 132, *r* = 0.49, *p* < 0.0001). The urine Hg levels also showed a significant positive correlation with hair levels (*n* = 132, *r* = 0.20, *p* = 0.0008). Notably, however, the blood Hg and urine Hg levels did not correlate significantly (*r* = 0.07, *p* = 0.35).

## 4. Discussion

RVs are routinely used to discover and compare populations with high environmental exposures to contaminants and in health-risk assessments, but to the best of our knowledge, no other recent study has compared the different methods of sampling from blood, urine, and hair to determine RVs in a group of potentially at-risk children. Our study calculated RVs as the 95th percentile, rounded off within the 95% confidence interval [5]. Although these values do not distinguish between hazardous and nonhazardous levels, they can be used statistically, to describe the body’s chemical load [18].

This is the first report that directly compared total Hg levels in blood, urine, and hair in children aged 0 to 6 years in Xiamen, China. Other studies in other countries have aimed to survey Hg exposure by analyzing Hg concentration in various samples (*i.e.*, urine, blood, and hair) and found levels similar to ours 1.05 μg/L (GM). For example, blood Hg levels averaged 0.94 μg/L (GM) in Ljubljana, Slovenia; GM 0.44 μg/L in Koprivnica, Croatia; 0.21 μg/L in Prague, Czech Republic; 0.12 μg/L in Wroclaw, Poland; 0.52 μg/L in Banska Bystrica, Slovakia and 0.43 μg/L in Landskrona, Sweden [19]. Blood Hg levels were higher in coastal areas than in inland areas, perhaps not surprisingly, in light of the idea that eating seafood increases blood Hg levels [20,21]. Our findings from this coastal city were in agreement with these data [22]. Very few previous studies analyzed their data according to sex but we identified whether our samples were collected from girls or boys. In our survey, for all sampling methods, boys and girls did not differ.

Urinalysis of our population showed a GM of 0.75 μg/L but our analysis did not correct for dilution. In contrast, a study conducted on 6-year-old children in 1991 by Walkowiak *et al.* [23] obtained urine Hg values of 0.16 μg /L (GM). The arithmetic mean found by Schulte *et al.* [24] in German 3- to 15-year-olds without amalgam fillings was 0.17 μg/L, which was lower than the arithmetic means in the present study (0.82 ± 0.30 μg/L). Again, in our study, the children’s urine Hg levels increased with growing age, with children aged 4 to 6 years showing significantly higher levels than those aged 0 to 1 years (*p* = 0.0096). Although urine mercury data was not normalized, we did collect morning samples, which may have had a normalizing influence. Indeed, our urinalysis data yield numbers consistent with expected outcomes.

In light of our findings, it is important to point out that currently the method of choice for evaluating an extended period of exposure to Hg is urinalysis. Our correlation analysis of blood and urine Hg levels showed that blood and urine Hg levels were not significantly related (*r* = 0.07, *p* = 0.35), while urine and hair correlation analysis yielded an *r* and *p* value of 0.20 and 0.0008, respectively. This result was in agreement with the study by Pesch *et al.* [25] that concluded hair and urine Hg levels correlated poorly (*r* = 0.297), and suggested analysis of hair cannot be considered an alternative method to the analysis of urine. Nevertheless, both methods contribute to a comprehensive estimation of the total Hg exposure; and our study did not analyze whether the Hg detected was organic or inorganic, analysis of urine is a better indicator for exposure to inorganic Hg, whereas analysis of hair is preferred for estimating the body’s burden of organic Hg compounds.

In our study of children’s hair, the GM value for Hg was 569.8 μg/kg, a result similar to that from a study of Inuit children, aged 3 to 5 years, who had a GM of 660 μg/kg, and another of 4-year old children from Granada (Spain) whose GM was 960 μg/kg [26]. Also, the analysis of hair in our study found older children exhibit a significantly higher Hg level. Similar positive associations between age and hair Hg levels were shown by Shao *et al.* [27]. Unlike the loose association of Hg levels in blood and urine, blood and hair levels showed a significant correlation (*r* = 0.49, *p* < 0.0001). This finding is in agreement with the result of Phelps *et al.* [28] who reported that Hg levels in hair and blood samples were linearly related.

## 5. Strengths and Limitations

The investigation is supported by several strengths. Our study examined a large group of children, including those 0 to 3 years. The literature contains very few such studies of children, and fewer still of very young children. In addition, we compared three methods of estimating mercury load, shedding light on the value of various sampling methods.

As for limitations, our urinalysis did not include normalization to creatinine level; however we did collect morning, spot-urine samples, which could enhance the consistency of our test results. Nevertheless, normalization to creatinine would be informative and in retrospect would be the optimal approach. In addition, the RVs reported here are specific for local population and not generalizable. The correlation of blood and urine with hair but not with each other is perplexing but might reflect that Hg can accumulate in organic and inorganic forms and is metabolized differently in various tissues.

## 6. Conclusions

We conducted a study for understanding Hg load in children in the coastal city of Xiamen, China, and calculated Hg RVs for blood at 2.30 μg/L, urine at 1.50 μg/L, and hair at 2100.0 μg/kg. Our results for blood and hair Hg levels exhibited a strong correlation (*r* = 0.49, *p* < 0.0001); and although Hg levels determined by analysis of urine and hair showed a significant positive correlation (*r* = 0.20, *p* = 0.0008), the Hg levels in blood did not correlate significantly with Hg levels in urine (*r* = 0.07, *p* = 0.35). We expect our findings will be informative for Hg risk assessment and management in children.
